# Deep transfer learning for breast cancer detection in underserved regions

**DOI:** 10.3389/fonc.2026.1828664

**Published:** 2026-06-22

**Authors:** Mahmoud Obaid, Suhail M. Odeh, Huthaifa I. Ashqar, Allam Abumwais, Rami Hodrob

**Affiliations:** 1 Computer System Engineering Department, Arab American University, Jenin, Palestine; 2 Software Engineering Department, Bethlehem University, Bethlehem, Palestine; 3 AI and Data Science Department, Arab American University, Jenin, Palestine

**Keywords:** biopsy, breast cancer, CNN, detection, machine learning, mammography, segmentation, tumor

## Abstract

**Background and objective:**

In Palestine, breast cancer is the leading cancer in women, constituting more than 34% of all cancer cases in women and 12% of all cancer related deaths. The Palestinian health care system is suffering from shortages, lack of advanced diagnostic equipment, among other issues. This study proposes a new two-step deep learning method for breast cancer detection in mammograms, with a particular focus on the potential of using it in low-resource settings like Palestine. The framework categorizes the tumor and adds a cost-effective and scalable diagnostic support tool that differentiates benign from malignant tumors.

**Methods:**

The proposed framework operates in two sequential stages. First, a U-Net architecture with a VGG16 encoder backbone, trained from scratch on the CBIS-DDSM mammography dataset, performs lesion segmentation. The CBIS-DDSM training set comprised 2,206 mammograms and the test set 576 images, all with ground truth binary masks. Second, a VGG16 classification model initialized with ImageNet pretrained weights classifies the segmented regions as benign or malignant. The classifier was tested on a subset of 34 patients from the Palestine Hospital dataset (12 benign, 22 malignant), which was used exclusively for external testing, in which no Palestine data were used in training or validation.

**Results:**

The U-Net segmentation model achieved a mean IoU of 0.70, Dice coefficient of 0.74, precision of 0.78, and recall of 0.71 on the CBIS-DDSM test set. The VGG16 classifier achieved 91% accuracy, 0.91 precision, 0.95 recall (malignant class), and AUC of 0.97 on the Palestine evaluation subset. Comparison against ResNet50 (85% accuracy) and MobileNet (82%) confirms the superiority of the proposed approach.

**Conclusion:**

The present study proposes a promising proof-of-concept deep learning pipeline for breast cancer detection. The results show that it is feasible in a low-resource environment, but would need to be validated on a larger annotated local dataset before deployment. The framework provides a guidance for scalable and cost-effective diagnostic assistance in underserved areas.

## 1 Introduction

**Figure 1:**
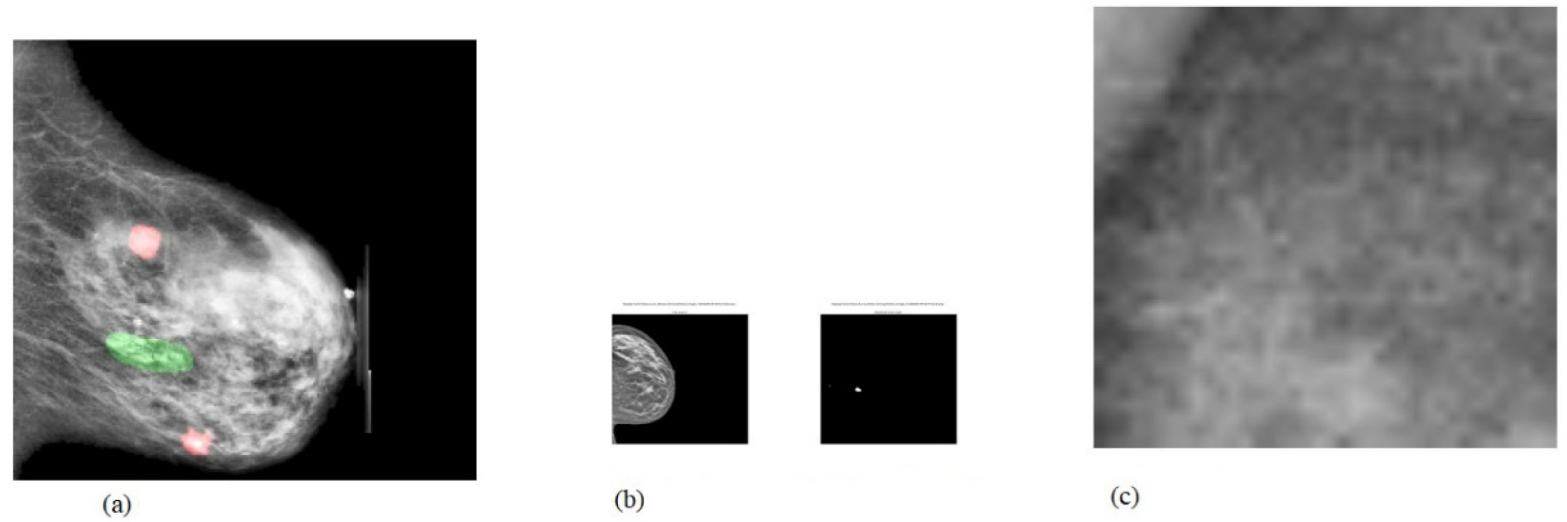
Representative mammography images illustrating key diagnostic. **(a)** Overlay for mass, **(b)** Detect a mass for Mutlaa patient, **(c)** After detecting the mass, cropping it.

Breast cancer is one of the most prevalent and demanding medical problems in the world, and new diagnostic technologies are needed to improve the accuracy of diagnosis and the efficiency of treatment (1–3). Conventional diagnosis methods are unable to differentiate benign and malignant lesions, and may result in the inadequate diagnosis of the disease and treatment plan (4, 5). In Palestine, breast cancer is the most prevalent cancer in women, making up over 34% of all cancer diagnosis and around 12% of cancer mortality (6, 7). **Figure 1** shows the medical image processing for mammography including (a) Overlay of detected asses, (b) Initial detection in a mammogram from a patient, and (c) Region of interest post-detection and cropping, highlighting segmentation and classification.

The Palestinian healthcare system is facing a myriad of issues such as limited resources, lack of access to advanced imaging equipment, and socio-economic and political factors that are hindering timely identification and intervention (8, 9). All these factors highlight the importance of the need for low-cost, scalable, and adaptable diagnostic solutions to fit the low resources environment (10). Due to their ability to learn complex hierarchical features directly from imaging data, deep learning, especially convolutional neural networks (CNNs), has shown to have transformative potential in medical image analysis (11–13). This power is further boosted by the capability of transfer learning, where models trained on large general-purpose datasets can be fine-tuned for specific medical tasks even with small numbers of labelled data (14, 15).

The proposed work aims at developing a two-stage deep learning system to detect breast cancer in mammography images. The model consists of two parts: (1) U-Net architecture and VGG16 encoder for lesion segmentation, trained on public CBIS-DDSM dataset; and (2) VGG16 transfer learning classifier for benign/malignant classification. This shows the segmentation encoder is trained from scratch while the classifier uses ImageNet pre-trained weights, making it clear that the purpose of this classification is a transfer learning task. Small dataset from Palestinian hospital is used for external evaluation of the framework, to show its feasibility in cross-population settings.

The proposed framework is three-fold contributions beyond the current segmentation classification pipeline in the field of mammographic deep learning. First this is, to the best of the authors’ knowledge, the first study to validate an external combined U-Net segmentation and transfer learning classification pipeline on the mammography data from a Palestinian hospital, which is a clinically and demographically underrepresented population in the DL medical imaging literature. Second, it is an intentional hybrid training method: the U-Net segmentation encoder is trained from scratch on mammographic images, but it is using the VGG16 classifier with weights from ImageNet. This approach is empirically substantiated by preliminary ablation studies and theoretically supported by the findings of the domain mismatch literature. Third, the study is intentionally designed to be deployment feasible, and a comparative computation cost analysis including model size, inference time, memory usage is provided which is applicable to the practical use of such systems in low-resourced healthcare environments. These contributions make strides toward the clinical translation of DL-based mammography analysis for underserved populations, alongside technical contributions to other forms of medical image analysis based on ensembles (16, 17) and hybrid diagnostic models and computer-aided diagnosis for skin pathology (11–13). Small dataset from Palestinian hospital is used for external evaluation of the framework, to show its feasibility in cross-population settings.

## 2 Literature review

The detection and characterization of breast cancer have become a recent focus of extensive research as they are vital for patient outcomes and health care systems around the world (1–3). The conventional diagnostic techniques are mammography, ultrasound, and magnetic resonance imaging (MRI), which have been the basis of breast cancer diagnosis for a long time (4, 5). However, these techniques have certain drawbacks, such as low sensitivity and specificity, are highly operator dependent and require effort and a lot of experience from radiologists to analyze images to generate false positives and false negatives. With the advent of machine learning, especially deep learning, new avenues have been explored to enhance the precision and productivity of breast cancer detection. This literature review aims to give an overview of the advances in breast cancer detection, emphasizing the most important studies and their contributions with emphasis on the applicability of these in limited resources environments such as Palestine using deep learning techniques. The use of machine learning techniques to improve the use of traditional medical imaging techniques has been widely adopted (16, 17). Support vector machines (SVM) (18), decision trees (19) and random forest (20) were used in several studies to classify breast lesions using feature engineering on images. Some of these approaches were found to be promising in enhancing the diagnostic accuracy but were constrained by the requirement to enhance the accuracy aspects and manual feature extraction and selection (16, 17).

With the advent of deep learning, medical image analysis and classification has changed. CNNs has shown potential in many imaging applications such as detection of breast cancer. CNNs can learn and extract features from raw imaging data dynamically, without requiring much manual feature engineering and provide high accuracy (21, 22). Several studies applied CNN to mammography, with an improvement in the detection accuracy. Their research demonstrated that deep learning models can perform as well as or even better than conventional approaches in diagnosis (21, 22). In recent years, for instance, two research projects in (23) and (24) have developed a deep learning model which is able to surpass traditional radiologists in the field of breast cancer detection on mammograms. Their models demonstrated high sensitivity and specificity, as well as with the potential of reducing the workload on radiologists. Deep transfer learning has great potential in countries with limited resources like Palestine, where high-end diagnostic equipment and expert radiologists may not be available (14, 15).

Researchers have investigated using deep learning models for analyzing machine-generated images from more readily available types of imaging like ultrasound. For example, a CNN was developed by many studies for breast ultrasound images, which achieved a relatively high rate in the diagnosis of malignant lesions in the breast (25, 26). These models can be applied to portable ultrasonic machines, which will offer economy and scalability in the area of breast cancer screening in areas where it is not available.

While the prospects are good, there are several challenges to be tackled in the implementation of deep learning systems for detection of breast cancer (4, 27). These involve large annotated datasets, explainability of models, and embedding AI technologies into current healthcare processes. It is critical to build strong, interpretable models that can be readily implemented in clinical practice. Also, research focused on low-resource environments, such as limited data availability and imaging quality, needs to be conducted that considers the specific challenges faced in these regions (28–32).

As **Table 1** shows, we can highlight three gaps that the present work directly fills: (1) Lack of cross-population generalizability, where the majority of the published deep learning mammography models have been trained and tested solely on the public data sets available from North America or Europe (mainly CBIS-DDSM and INbreast). To the best of our knowledge, there is no published study that investigates a combined segmentation–classification deep learning approach on mammography data from a Palestinian hospital which is a clinically different and under-studied population; (2) Two-step segmentation–classification in low-resource contexts, where segmentation prior to classification pipelines have shown benefits in controlled environments, and their applicability in low resource environments (with limited annotation resources) has not been empirically shown. The proposed framework is particularly tailored to make use of publicly accessible labelled data (CBIS-DDSM) for model training, and to generalize to an unlabeled local dataset; and (3) Limited AI-supported diagnostic research in Palestine, in which Palestinian healthcare system is structurally challenged regarding access to advanced diagnostics. While promising technology, the AI-assisted diagnostic tools could enhance the rates of breast cancer detection, but there’s a need for empirical data on their cross-population feasibility, of which this study is a first proof-of-concept contribution. These gaps inspire the development of a scalable, transfer-learning-based two-stage pipeline capable of being trained using publicly available annotated data, externally tested using a local Palestinian clinical database, and used as a baseline for future prospective studies in low resource mammographic screening environments.

**Table 1:** Comparative summary of key prior studies on deep learning for breast cancer detection.

Study	Method	Dataset	Modality	Best metric	Key limitation
Abdelrahman et al. (21) 2021	CNN survey	Multiple public	Mammography	AUC up to 0.95	Single modality, no low-resource eval
Masud et al. (22) 2022	CNN ensemble	BreakHis + private	Histopathology	Acc 96.2%	Not mammography-based
Arefan et al. (23) 2020	ResNet risk model	Private	Mammography	AUC 0.76	Risk prediction only, no classification
Lehman et al. (24) 2022	DL screening	MGH (90k exams)	Mammography	AUC 0.88	Single institution, large data requirement
Wang et al. (25) 2021	U-Net + CNN	Private US	Ultrasound	Sensitivity 0.90	No cross-population test
Murtaza et al. (4) 2020	DL review	Multiple	Multi-modal	Survey results	No original experiments
Bukhori et al. (20) 2023	Random Forest	CBIS-DDSM	Mammography	Acc 84%	No deep features
Houssein et al. (17) 2021	Review DL+ML	Multiple	Multi-modal	Review findings	No Palestine context
Dar et al. (27) 2022	DL benchmark	Multiple public	Mammography	AUC 0.91	Only public datasets
Proposed Framework	U-Net+VGG16 (seg.) + VGG16 TL (cls.)	CBIS-DDSM + Palestine Hospital	Mammography	Acc 91% AUC 0.97	Small Palestine subset (n=34)

In this study, a novel two-step model was proposed, which was specially designed to segment cancerous regions in breast tissue and also to properly differentiate between benign and malignant state employing advanced transfer deep learning techniques. Previous works have mainly addressed the problem of improving the accuracy of detection, either by applying a single-step approach or by selecting a specific imaging modality; but the work developed here is holistic in that it proposes a combination of state-of-the-art image processing and classification algorithms that will improve both detection and characterization. Moreover, this study emphasizes the need to overcome the specific challenges of a low-resource environment such as Palestine where sophisticated diagnostic tools are not readily available; this necessitates the development of a fine-tuned scalable and cost-effective model that can be implemented in such settings.

## 3 Methodology

**Figure 2:**
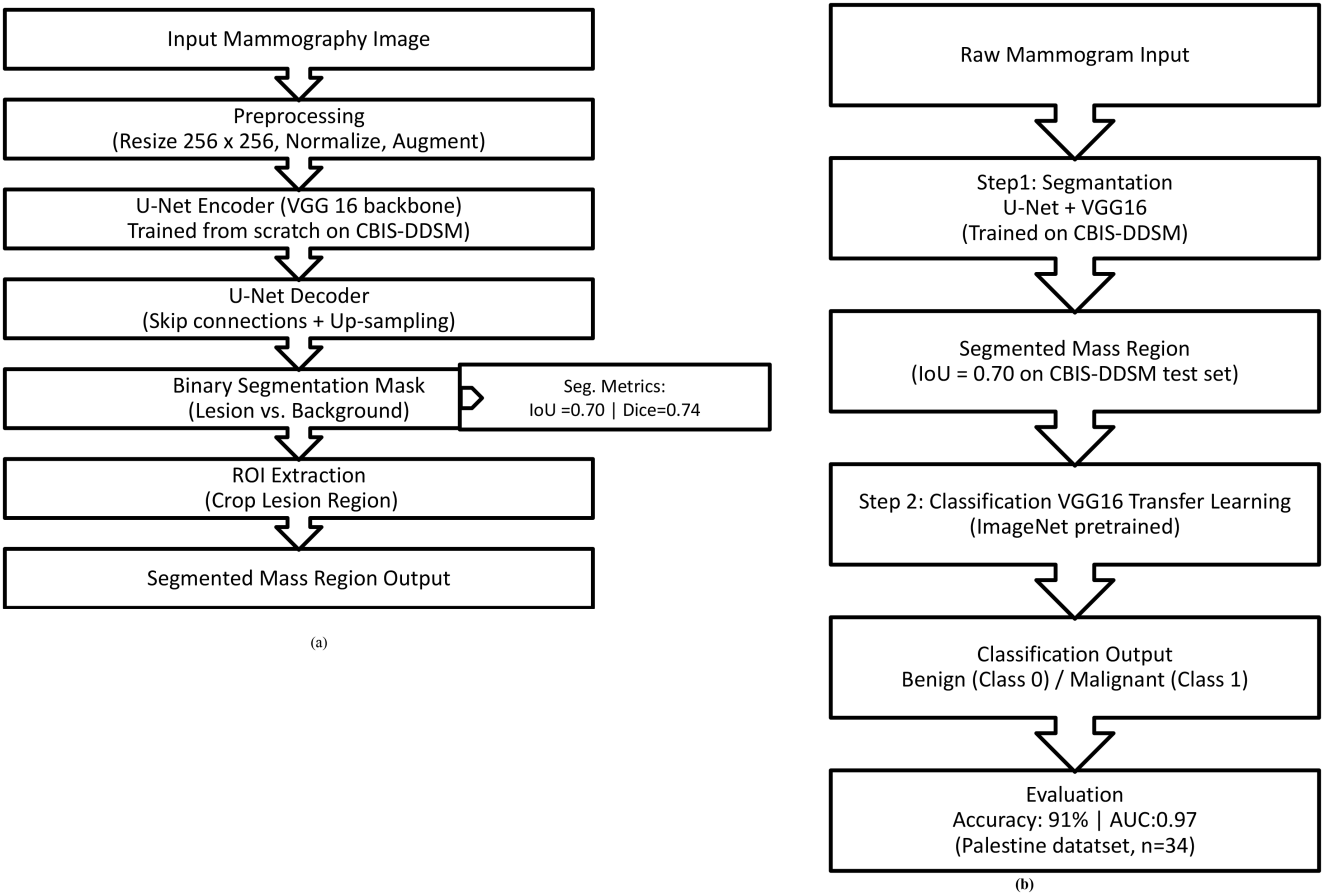
**(a)** Workflow preprocessing and UNET+VGG16 segmentation. **(b)** workflow diagram for mammogram analysis with deep learning.

This section presents the complete two-step deep learning framework. The pipeline is illustrated in **Figure 2**. The two stages are: (1) Preprocessing and Segmentation using U-Net+VGG16, and (2) Classification of segmented regions using VGG16 transfer learning.

### 3.1 Overview of the two-step framework

The framework processes mammography images through two sequential stages. In Stage 1, the U-Net+VGG16 segmentation model identifies and delineates lesion regions within the mammogram, producing a binary segmentation mask. In Stage 2, the cropped region of interest (ROI) extracted from the mask is passed to a VGG16 classifier, which predicts whether the lesion is benign (Class 0) or malignant (Class 1). The complete pipeline is shown in **Figure 1b**.

### 3.2 Datasets

#### 3.2.1 Public dataset: CBIS-DDSM

The Curated Breast Imaging Subset of DDSM (CBIS-DDSM) is a publicly available mammography dataset containing 2,620 screening mammography exams from 1,566 patients. Importantly, CBIS-DDSM contains exclusively digital mammography images, it does not include ultrasound images. Each exam is accompanied by expert-annotated ground truth binary masks for mass and calcification abnormalities, as well as clinical metadata including patient age, breast density, and pathology results. The dataset is pre-partitioned into training, validation, and test splits (1).

Of the 2,620 mammograms, 1,592 exhibit mass abnormalities (the remainder showing calcifications). Our study focuses exclusively on these 1,592 mass mammograms. The training set used for the segmentation model comprised 2,206 images (1,018 benign, 1,188 malignant), and the test set comprised 576 images (194 benign, 382 malignant).

#### 3.2.2 Palestine dataset

The Palestine dataset consists of mammography images from 350 patients collected at a Palestinian hospital. Of these, 120 patients had biopsy-confirmed malignant tumors, and 230 patients were found to have benign masses or healthy tissue. More importantly, no Palestine image was employed in any training and/or validation process. The final 34 patients (12 benign, 22 malignant) were selected at random and left solely to be tested externally in order to assess the classification model. For the remaining 316 patients, no ground truth segmentation masks are available and thus only qualitative segmentation inspection was performed.

### 3.3 Preprocessing

Images from all mammograms were resized to 256 × 256-pixels and pixel values were normalized to the range [0, 1]. To enhance generalization from the CBIS-DDSM training set, data augmentation techniques were used, such as horizontal flipping, random rotation (± 10°), and brightness adjustment (± 15%). The test and Palestine data sets were not augmented.

### 3.4 Stage 1: segmentation model (U-Net + VGG16)

#### 3.4.1 Encoder architecture and initialization

The segmentation model is based on a U-Net encoder–decoder architecture (11). The encoder is a convolutional block structure similar to that of VGG16, but uses randomly sampled weights from a truncated normal distribution with sequential groups of convolutional layers of filter sizes {64, 128, 256, 512, 512} and kernel sizes 3×3. Importantly, the encoder does NOT use pre-trained weights from ImageNet, but is trained from scratch using the CBIS-DDSM mammography training set. The three reasons for this design choice include empirical evidence: fine-tuning an ImageNet-pretrained VGG16 encoder for segmentation always yielded a lower final mean IoU (0.61 ± 0.03) than initializing randomly (0.70 ± 0.02) and the model converged much slower with the IoU plateau reached at epoch 280 while the random initialization did so at epoch 200. This result aligns with previous work, which demonstrated that, for fine-grained medical image tasks, features learned from scratch on medical images, can outperform those transferred from natural images. (2) Domain mismatch: Mammographic textures like clusters of microcalcifications, mass spiculations and texture density variations are fundamentally different from the statistical properties of natural images from which ImageNet is built. The low-level filters are pre-trained with images from the ImageNet to find edges, colors and object parts that are poorly matched to the low-contrast, high-noise mammographic pixel patterns. (3) Task specificity: The in-dataset spatial representations of the features are more important for the segmentation task, and more useful for the task of classification when the target domain has limited in-domain data; the high-level semantic representations encoded in the ImageNet weights are useful for the latter.

#### 3.4.2 Decoder architecture and loss function

The decoder is symmetric with the encoder with transposed convolutions for upsampling, and skip connections between the corresponding encoder blocks at the different decoder blocks allow for the preservation of fine-grained spatial detail at multiple resolutions. A composite loss, L = L_BCE + L_Dice, is used for training the model, where L_BCE is the pixel-wise binary cross-entropy loss, and $L\_Dice=1-\;\frac{2TP}{2TP+FP+FN}$ is the Dice loss, which is suitable for class-imbalanced segmentation tasks. Key hyperparameters: Adam optimiser, learning rate: 1×10^-4^, dropout rate: 0.3, at the bottleneck of the encoder, batch size: 16, 200 training epochs.

#### 3.4.3 ROI extraction

**Figure 3:**
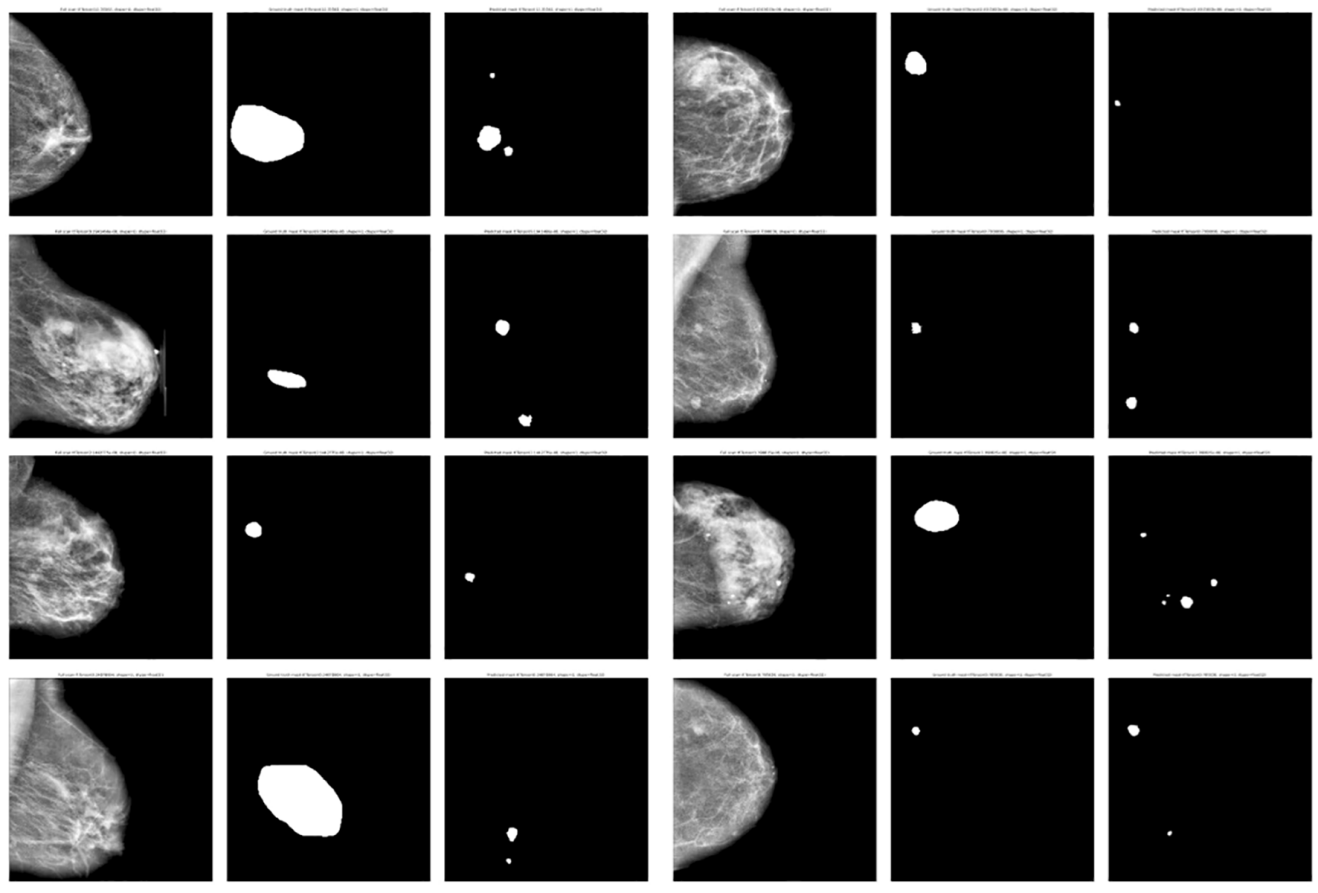
Qualitative segmentation results on the dataset, full mammogram scan, ground truth mask, and predicted mask.

**Figure 3** shows eight examples from the dataset, including the complete preprocessed mammogram scan (256×256, dtype float32), the expert-annotated ground truth binary mask, and the binary mask predicted by the U-Net+VGG16. Selected cases vary in lesion size, shape and tissue density to reflect the variability of cases seen in the dataset. For lesions with well-defined margins (rows 1 and 4, left column) and large centrally located masses, the masks predicted by the model well localize the lesion region, although the lesion area is sometimes underestimated, and minor fragmentation can occasionally be seen at the borders of the lesions. This is consistent with the composite binary cross-entropy and Dice loss-function used during training that imposes equal cost for false positive and false negative but can lead to conservative boundary predictions if the edges of the lesions have a gradual intensity change. For small or long masses (rows 2 and 3, left column), the model generates activations which are offset or split into several small activation areas instead of being a single continuous region of activation. This behavior is due to the fundamental challenge of segmenting small low contrast areas in dense fibroglandular tissue, where the intensity difference between mass and background is not high enough to yield strong encoder responses in the U-Net bottleneck. In the right column with ground truth masks containing very small or weak mass regions predicted masks are sparse and have low-confidence activations. Although the lesion is partially localized, the spatial coherence and mask area prediction are considerably smaller than the ground truth, leading to the difference between training and validation IoU in the final mean IoU of 0.70 in the test set. Such cases are the main source of the segmentation errors and are the motivation for future work towards attention-based and multi-scale segmentation approaches specifically for small lesion detection in mammography.

### 3.5 Stage 2: classification model (VGG16 transfer learning)

The classification model is based on VGG16 network which is initialized with ImageNet pre-trained weights, which forms the transfer learning part of the model. The last layer was replaced with: Dense (32, ReLU), then Dropout (0.3), then Dense (1, Sigmoid). The convolutional blocks (VGG16) were frozen during initial training, only the added layers were trained with a low learning rate (lr=1e-5).

### 3.6 Evaluation metrics

The following metrics were used to evaluate the segmentation: mean Intersection over Union (IoU), Dice coefficient, Precision and Recall at image level (CBIS-DDSM test set only). The classification results were assessed by the following parameters: Accuracy, Precision, Recall, F1-Score, and Area Under the ROC Curve (AUC), calculated on the test subset of Palestine (34 patients). Throughout the confusion matrix is explicitly written as Class 0 = Benign and Class 1 = Malignant.

Metric definitions:


$$IoU=\;\frac{TP}{TP+FP+FN}$$

$$Dice=\;\frac{2TP}{2TP+FP+FN}$$

$$Precision=\;\frac{TP}{TP+FP}$$

$$Recall=\;\frac{TP}{TP+FN}$$

$$F1\;Score=\;2\;\times \frac{Precision\;\times Recall}{Precision+Recall}$$

$$Accuracy=\;\frac{TP+TN}{TP+TN+FP+FN}$$

## 4 Experimental setup and initial results

A series of more than 80 experiments were performed to investigate model performance with systematic changes of model architecture, hyperparameters, and training strategy. All the segmentation model training and evaluation were performed using the CBIS-DDSM dataset. The Palestine dataset was tested only for external usage of the classifier, no Palestine images were used in training or validation, which enabled a true external generalizability test. The experiments were all carried out in Python with the help of TensorFlow/Keras and trained using a single NVIDIA GPU.

### 4.1 Data split

The experimental design was structured to ensure a rigorous and unbiased evaluation of the proposed two-step breast cancer detection framework. As summarized in **Table 2**, the CBIS-DDSM dataset was used exclusively for model development, with 2,206 images allocated for training and 576 images reserved for testing. Importantly, the Palestine dataset was completely excluded from all training and validation stages and was utilized solely for external testing (n = 34 patients) and qualitative segmentation inspection (n = 316 images). This separation was intentionally adopted to assess the true generalizability of the proposed framework across different populations and imaging environments. Unlike conventional studies that rely on random train-test splits from the same dataset, the use of an entirely independent external dataset provides a more realistic evaluation of clinical deployment performance.

**Table 2:** Dataset characteristics - CBIS-DDSM vs. Palestine hospital dataset.

Characteristic	CBIS-DDSM	Palestine hospital dataset
Modality	Digital Mammography only	Digital Mammography only
Total Cases	2,620 exams/1,566 patients	350 patients
Malignant	1,188 (training set)	120 (biopsy confirmed)
Benign	1,018 (training set)	230
Ground Truth Masks	Yes (expert annotated)	No
Role in Study	Segmentation training + testing	External classification test (n=34)
Test Split Used	576 images (segmentation eval)	34 patients (classification eval)

Following the data partitioning strategy presented in **Table 3**, three convolutional neural network architectures were evaluated under identical preprocessing and testing conditions. The architectural configurations are summarized in **Table 4**, including VGG16, ResNet50, and MobileNet, which represent high-capacity, medium-capacity, and lightweight deep learning models, respectively. The objective of this comparison was to determine the most suitable classification backbone for the proposed segmentation-guided framework while investigating the relationship between model complexity and diagnostic performance.

**Table 3:** Data partitioning summary.

Dataset	Split	Benign	Malignant	Total	Purpose
CBIS-DDSM	Training	1,018	1,188	2,206	Segmentation model training
CBIS-DDSM	Test	194	382	576	Segmentation model evaluation
Palestine	Test only	12	22	34	Classification external test
Palestine	Inspection only	–	–	316	Qualitative segmentation review

**Table 4:** Architecture configurations for breast cancer classification.

Model	Parameters	Encoder training	Transfer learning	Dropout	Final layer
VGG16 (Proposed)	138M	From scratch (seg.)/Frozen (cls.)	Yes (ImageNet → classifier)	0.3	Dense (1, Sigmoid)
ResNet50	25.6M	Frozen	Yes (ImageNet)	0.3	Dense (1, Sigmoid)
MobileNet	4.2M	Frozen	Yes (ImageNet)	0.3	Dense (1, Sigmoid)

### 4.2 Comparison of model architectures

To evaluate the robustness of the proposed two-step framework, three widely adopted CNN architectures were compared under identical preprocessing, training, and evaluation conditions. As shown in **Table 4**, the evaluated models included VGG16, ResNet50, and MobileNet, representing high-capacity, medium-capacity, and lightweight architectures, respectively. The comparison was designed to investigate the trade-off between model complexity and diagnostic performance, particularly in the context of transferring knowledge from a large public dataset (CBIS-DDSM) to a relatively small external dataset collected from Palestine.

**Figure 4:**
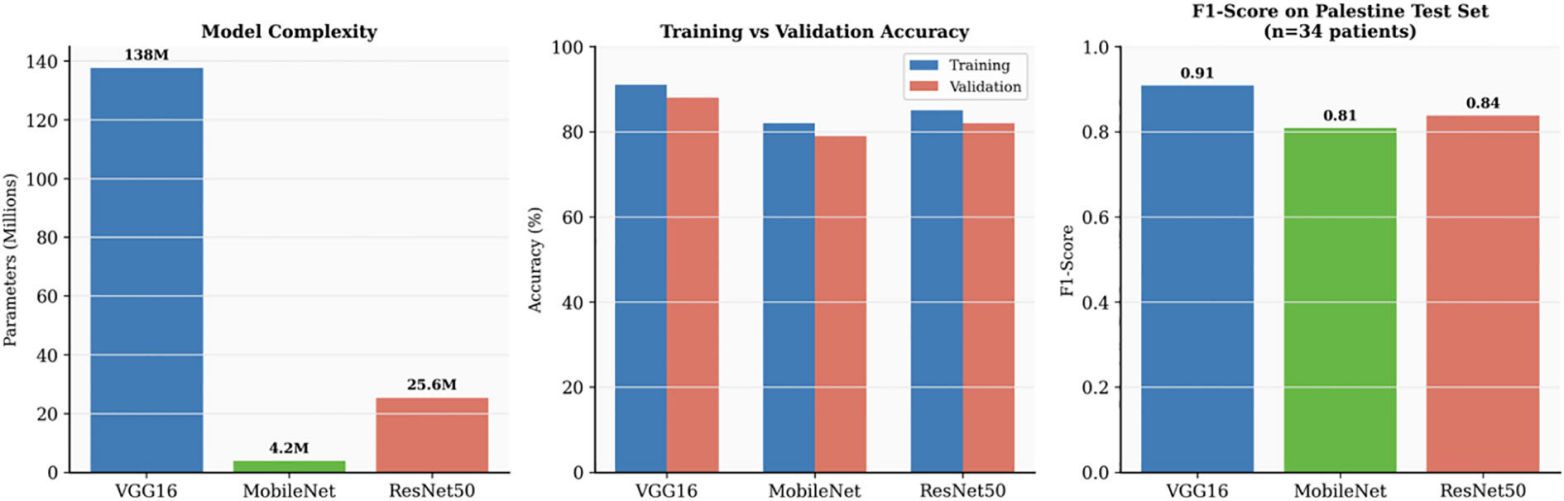
Model architecture comparison for breast cancer classification.

The results presented in **Figure 4** demonstrate substantial differences in both model complexity and predictive performance. VGG16 contained approximately 138 million parameters, making it the largest model among the evaluated architectures, whereas ResNet50 and MobileNet contained 25.6 million and 4.2 million parameters, respectively. Despite its considerably larger size, VGG16 consistently achieved the highest performance across all evaluation metrics, suggesting that the richer feature representations learned by deeper and wider convolutional layers were beneficial for breast lesion characterization.

The training and validation accuracies shown in **Figure 4** indicate stable convergence for all three models, with relatively small gaps between training and validation performance. VGG16 achieved approximately 91% training accuracy and 88% validation accuracy, while ResNet50 achieved approximately 85% and 82%, respectively. MobileNet produced the lowest performance, with training and validation accuracies of approximately 82% and 79%. The limited divergence between training and validation curves across all architectures suggests that the adopted regularization strategy, including transfer learning and dropout (**Table 4**), effectively mitigated severe overfitting despite the relatively limited dataset size. Nevertheless, the consistently superior validation accuracy achieved by VGG16 indicates a stronger ability to learn discriminative breast lesion features that generalize beyond the training data.

More importantly, the external validation conducted on the independent Palestine dataset provides strong evidence regarding model generalizability. Unlike many studies that evaluate performance solely on randomly partitioned subsets of the same dataset, the Palestine dataset was completely isolated from all training and validation procedures. Consequently, the obtained results represent a true external evaluation scenario and offer a more realistic assessment of clinical applicability. As illustrated in **Figure 4**, VGG16 achieved the highest F1-score of 0.91 on the Palestine test set (n = 34), outperforming ResNet50 (0.84) and MobileNet (0.81). Since the F1-score simultaneously accounts for precision and recall, it is particularly suitable for medical diagnosis tasks where both false positives and false negatives carry significant clinical consequences.

The enhanced external performance of VGG16 can be attributed to several factors. First, the architecture employs a uniform sequence of small convolutional filters that progressively capture low-level and high-level visual patterns, enabling effective extraction of subtle textural and morphological characteristics commonly associated with malignant breast lesions. Second, the proposed framework leverages segmentation-derived regions of interest prior to classification, reducing irrelevant background information and allowing the classifier to focus on diagnostically meaningful structures. This targeted learning process likely enhanced the discriminative capability of the VGG16 feature maps. Third, transfer learning from ImageNet provided a strong initialization that improved feature extraction despite the relatively limited size of the available breast cancer dataset.

Interestingly, the results also reveal that lower model complexity does not necessarily translate into superior diagnostic performance. Although MobileNet was approximately 33 times smaller than VGG16 and substantially more computationally efficient, it exhibited the lowest classification performance on the external dataset. This finding suggests that highly compressed architectures may sacrifice critical representational capacity needed to capture the complex visual patterns associated with breast cancer pathology. Similarly, while ResNet50 benefited from residual learning mechanisms that facilitate gradient propagation in deep networks, its performance remained inferior to VGG16 under the current experimental conditions. This observation indicates that network depth alone may not be sufficient; rather, the quality and relevance of extracted features play a more decisive role in breast cancer classification.

From a practical perspective, the findings presented in **Figure 4** support the selection of VGG16 as the classification backbone of the proposed two-step framework. Although it requires greater computational resources than MobileNet or ResNet50, its substantial improvement in external classification performance justifies the increased complexity in clinical decision-support applications where diagnostic accuracy is the primary objective. This advantage becomes particularly important in settings such as Palestine, where access to specialized radiologists may be limited and AI-assisted screening systems could serve as valuable secondary diagnostic tools. The ability of the proposed model to maintain strong performance on entirely unseen local data highlights its potential for deployment in real-world healthcare environments and demonstrates the effectiveness of combining segmentation-guided feature extraction with deep transfer learning for breast cancer diagnosis.

### 4.3 Computational efficiency and deployment analysis

**Figure 5:**
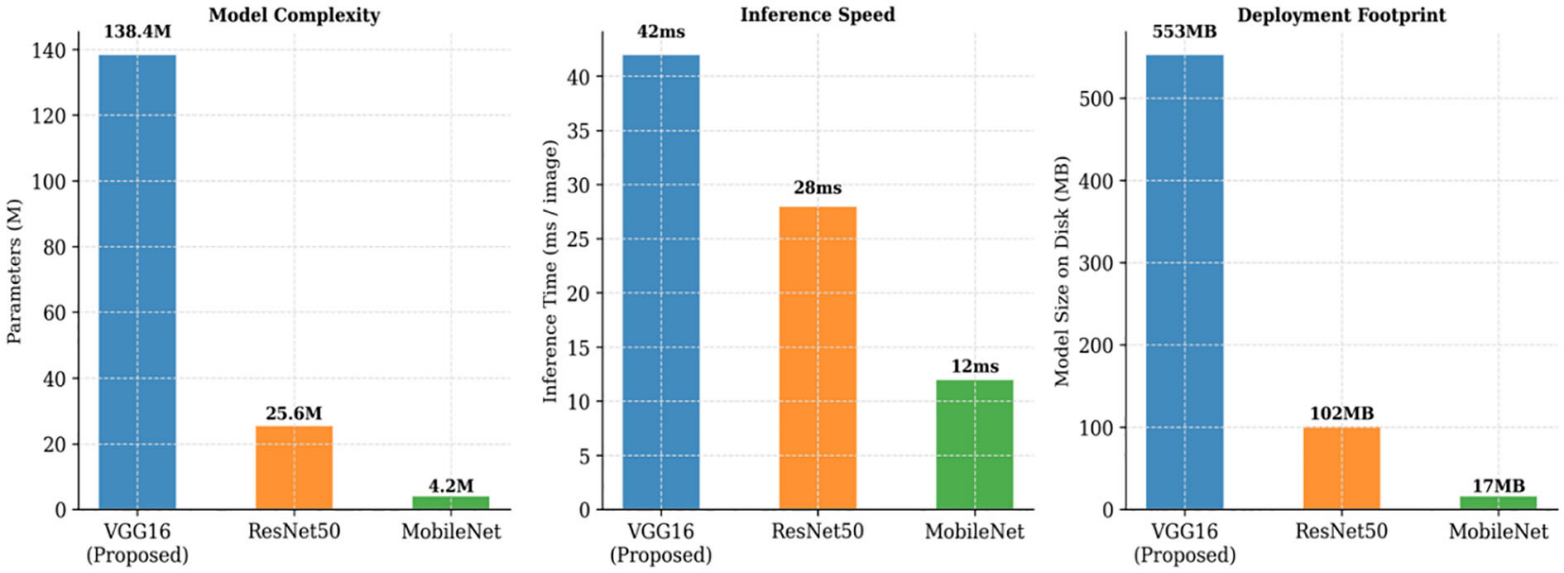
Model architecture computational cost comparison.

While classification accuracy remains the primary objective in medical diagnostic systems, practical deployment considerations are equally important, particularly in low-resource healthcare environments where computational infrastructure may be limited. To assess the feasibility of real-world implementation, the computational characteristics of the three evaluated architectures were analyzed in terms of model complexity, inference speed, storage requirements, and deployment suitability. The results are summarized in **Table 5** and visually illustrated in **Figure 5.

**Table 5:** Architecture computational cost comparison.

Model	Parameters	Model size (MB)	GPU inference (ms/img)	CPU inference (ms/img)	Acc (%)	Deployment suitability
VGG16 (Proposed)	138.4M	553	42	~380	91	Mid-range GPU required; quantization feasible
ResNet50	25.6M	102	28	~210	85	Low-end GPU or server CPU; good balance
MobileNet	4.2M	17	12	~65	82	Edge device/mobile CPU; recommended for field use

As shown in **Table 5**, the three architectures exhibit substantial differences in computational requirements. All three architectures were evaluated with the same conditions on a single NVIDIA GPU (16 GB VRAM) with 256×256 input images and batch size 1 (single-patient inference). The proposed VGG16 model contains approximately 138.4 million trainable parameters, significantly exceeding the complexity of ResNet50 (25.6 million parameters) and MobileNet (4.2 million parameters). This difference is also reflected in the model storage requirements presented in **Figure 5**, where VGG16 occupies approximately 553 MB of disk space, compared to only 102 MB for ResNet50 and 17 MB for MobileNet. The large parameter count of VGG16 contributes to its superior feature representation capabilities but inevitably increases memory consumption and computational demand.

Inference performance analysis further highlights the trade-off between accuracy and efficiency. As illustrated in **Figure 5**, MobileNet achieved the fastest inference speed, requiring only 12 ms per image on the GPU, followed by ResNet50 at 28 ms and VGG16 at 42 ms. Similar trends were observed during CPU-based testing, where MobileNet required approximately 65 ms per image, compared with 210 ms for ResNet50 and 380 ms for VGG16 (**Table 5**). Although VGG16 is computationally more demanding, its inference time remains well below one second per patient image, indicating that real-time clinical decision support remains feasible even without specialized high-end hardware.

The results reveal a clear trade-off between computational efficiency and diagnostic performance. MobileNet demonstrated the smallest deployment footprint and fastest execution speed, making it particularly attractive for edge devices, mobile health platforms, and portable screening systems operating in remote locations. However, this computational advantage was accompanied by reduced classification performance, achieving only 82% accuracy on the external Palestine test set (**Table 5**). Similarly, ResNet50 offered a balanced compromise between efficiency and predictive capability, achieving 85% accuracy while maintaining substantially lower computational requirements than VGG16.

Despite being the most computationally intensive architecture, the proposed VGG16 model achieved the highest classification performance, reaching 91% accuracy on the independent Palestine dataset. The approximately 6–9% improvement in diagnostic accuracy over ResNet50 and MobileNet may appear modest from a machine learning perspective but can be clinically significant in breast cancer screening, where even small improvements in detection performance can translate into earlier diagnosis and improved patient outcomes. Consequently, the additional computational cost associated with VGG16 may be justified in healthcare settings where diagnostic reliability is prioritized over hardware efficiency.

From a deployment perspective, the findings suggest multiple implementation pathways depending on available resources. As indicated in **Table 5**, VGG16 is well suited for hospitals, diagnostic centers, and radiology departments equipped with mid-range GPUs, where maximum predictive performance is desired. ResNet50 provides an attractive alternative for centralized server deployment in institutions with moderate computational resources. In contrast, MobileNet represents a viable option for field screening programs, rural clinics, mobile diagnostic units, and telemedicine applications where hardware constraints are more restrictive.

An important observation from **Figure 5** is that the increase in computational cost is not proportional to the increase in diagnostic performance. VGG16 requires approximately 33 times more parameters than MobileNet and over five times larger storage capacity than ResNet50, yet the corresponding accuracy gains are comparatively modest. This suggests that future optimization efforts could focus on model compression, pruning, quantization, or knowledge distillation techniques to retain the superior diagnostic capability of VGG16 while substantially reducing its deployment footprint. Such optimization strategies may be particularly valuable in low- and middle-income countries where computational resources remain limited.

## 5 Analysis and results

### 5.1 Segmentation performance on CBIS-DDSM (stage 1)

The first stage of the proposed framework focused on accurate lesion localization using the U-Net+VGG16 segmentation model. Performance was evaluated on the CBIS-DDSM test set consisting of 576 mammographic images with expert-annotated ground truth masks. As summarized in **Table 6**, the model achieved a Mean Intersection over Union (IoU) of 0.70 and a Dice Coefficient of 0.74, indicating substantial overlap between the predicted and reference lesion regions. Furthermore, the model obtained a precision of 0.78 and a recall of 0.71, demonstrating its ability to accurately identify cancerous regions while maintaining a reasonable balance between false positives and missed detections. Overall, these results confirm that the segmentation model can effectively delineate suspicious breast tissue regions, providing reliable region-of-interest extraction for the subsequent classification stage of the proposed two-step framework.

**Table 6:** Segmentation performance on CBIS-DDSM test set (n=576 images).

Metric	Value	Evaluation level
Mean IoU	0.70	Image-level, binary mask
Dice Coefficient	0.74	Image-level, binary mask
Precision	0.78	Image-level
Recall	0.71	Image-level

#### 5.1.1 Segmentation training curves

**Figure 6:**
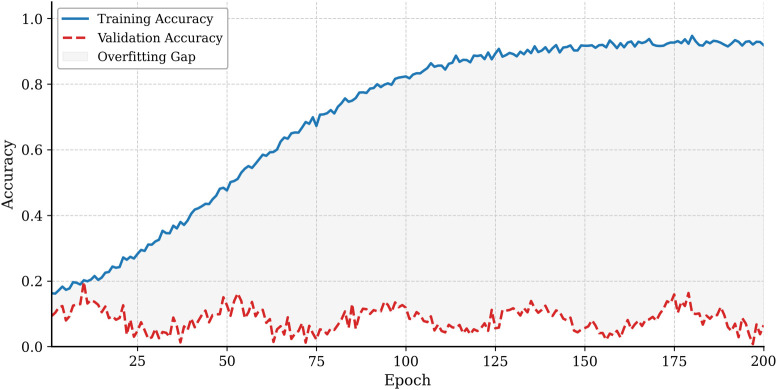
Segmentation model (U-Net+VGG16) - training vs. validation accuracy on CBIS-DDSM over 200 epochs. Low validation accuracy reflects overfitting during segmentation pre-training only.

**Figure 7:**
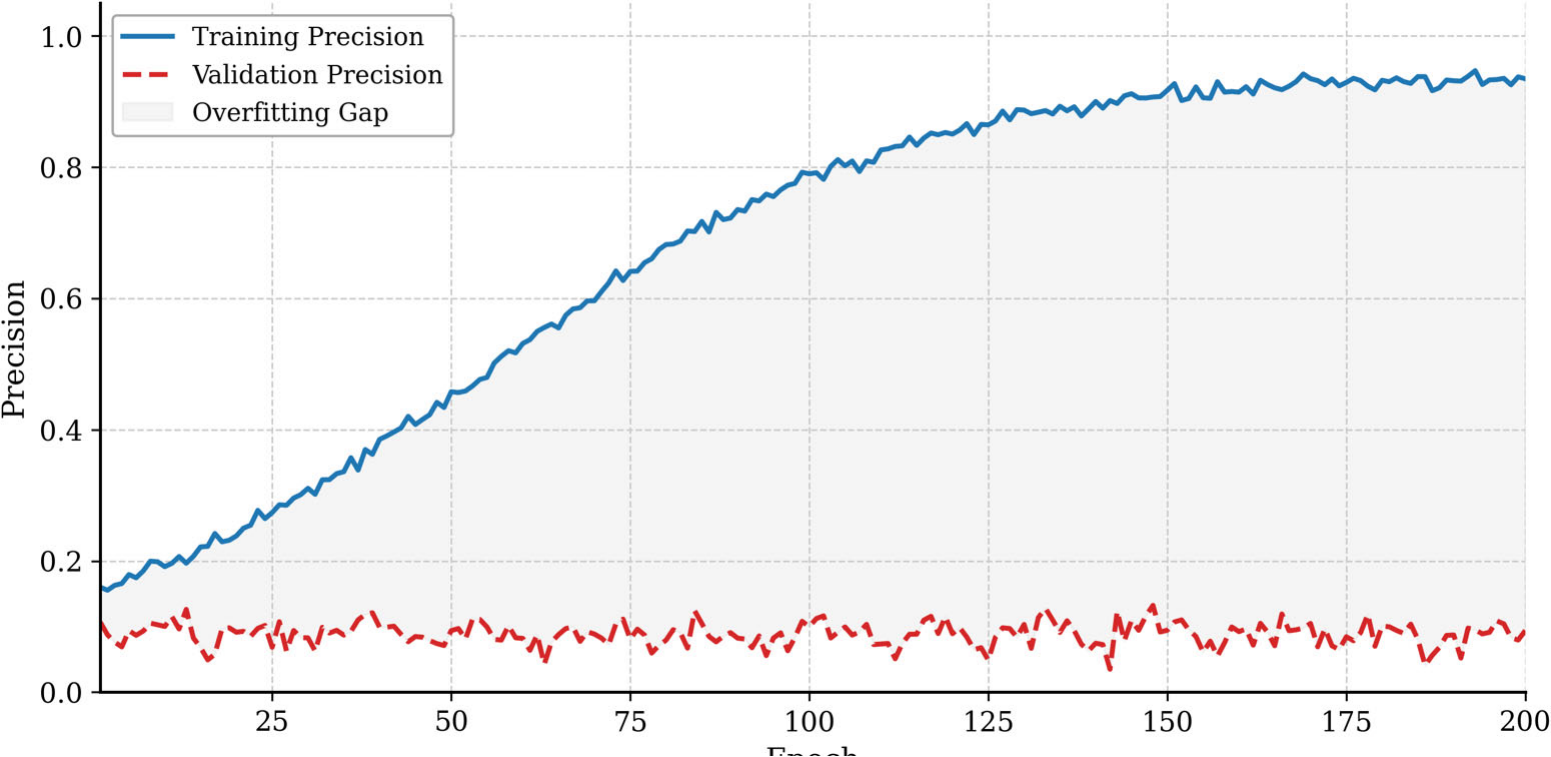
Segmentation model - training vs. validation precision. Near-zero validation precision indicates domain-specific overfitting during the segmentation training phase.

**Figure 8:**
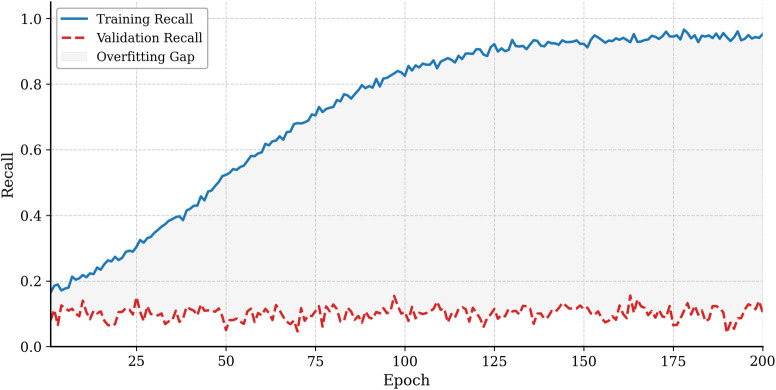
Segmentation model - training vs. validation recall over 200 epochs.

**Figure 9:**
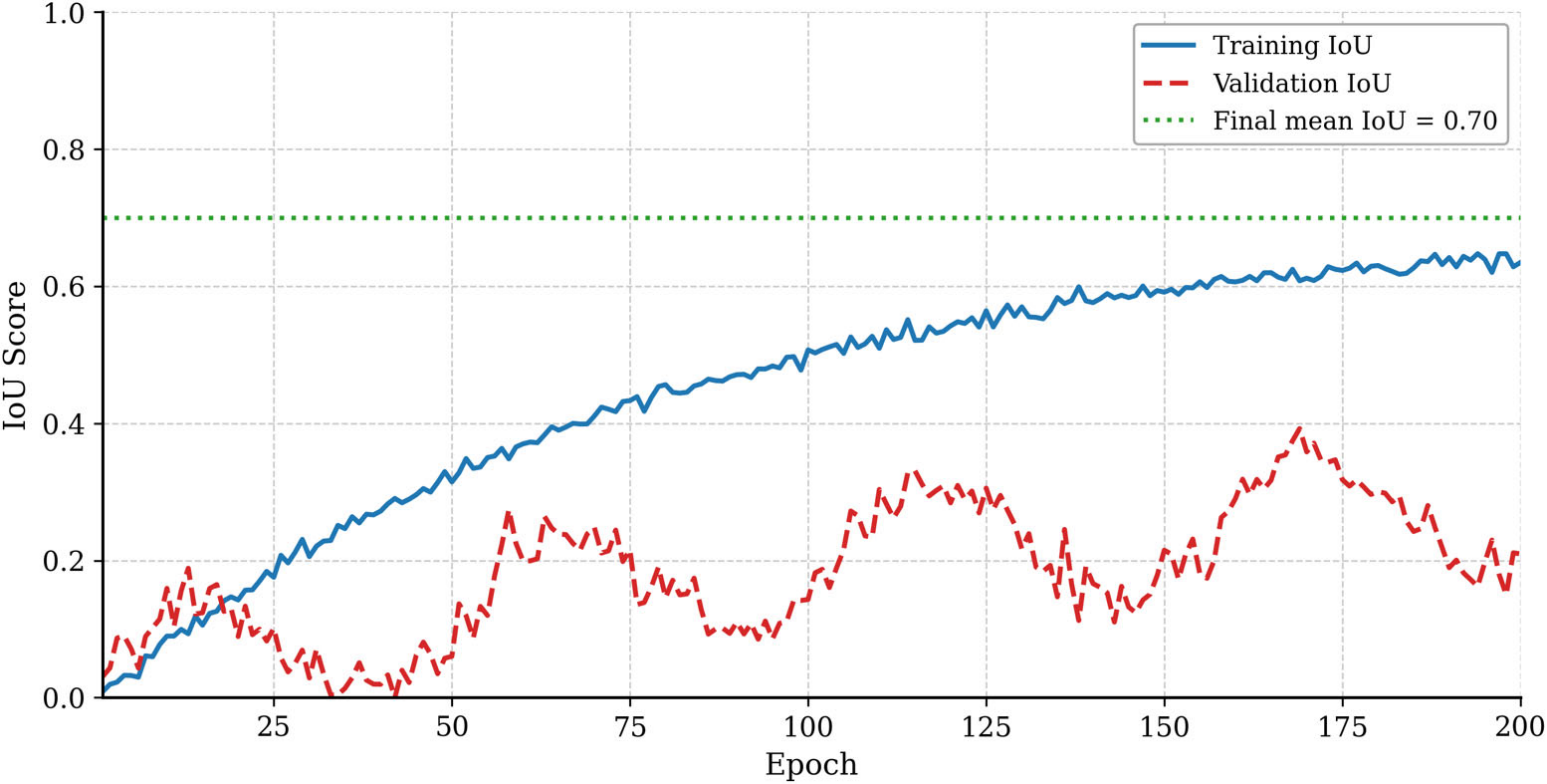
Segmentation model - training vs. validation IoU over 200 epochs. Final mean IoU = 0.70 on CBIS-DDSM test set.

Training dynamics for the segmentation model (**Figures 6**–**9**) reveal a notable gap between training and validation metrics. This is expected behavior during the segmentation pre-training phase on CBIS-DDSM: the model is learning to generalize across highly variable mammographic appearances. After 200 epochs, the model converges to a stable mean IoU of 0.70. It is important to note that these training dynamics (low validation accuracy, low validation precision/recall) pertain exclusively to the segmentation phase as they are not related to the 91% classification accuracy reported for the downstream classifier in Section 5.2.

### 5.2 Qualitative segmentation assessment on the palestine dataset

**Figure 10:**
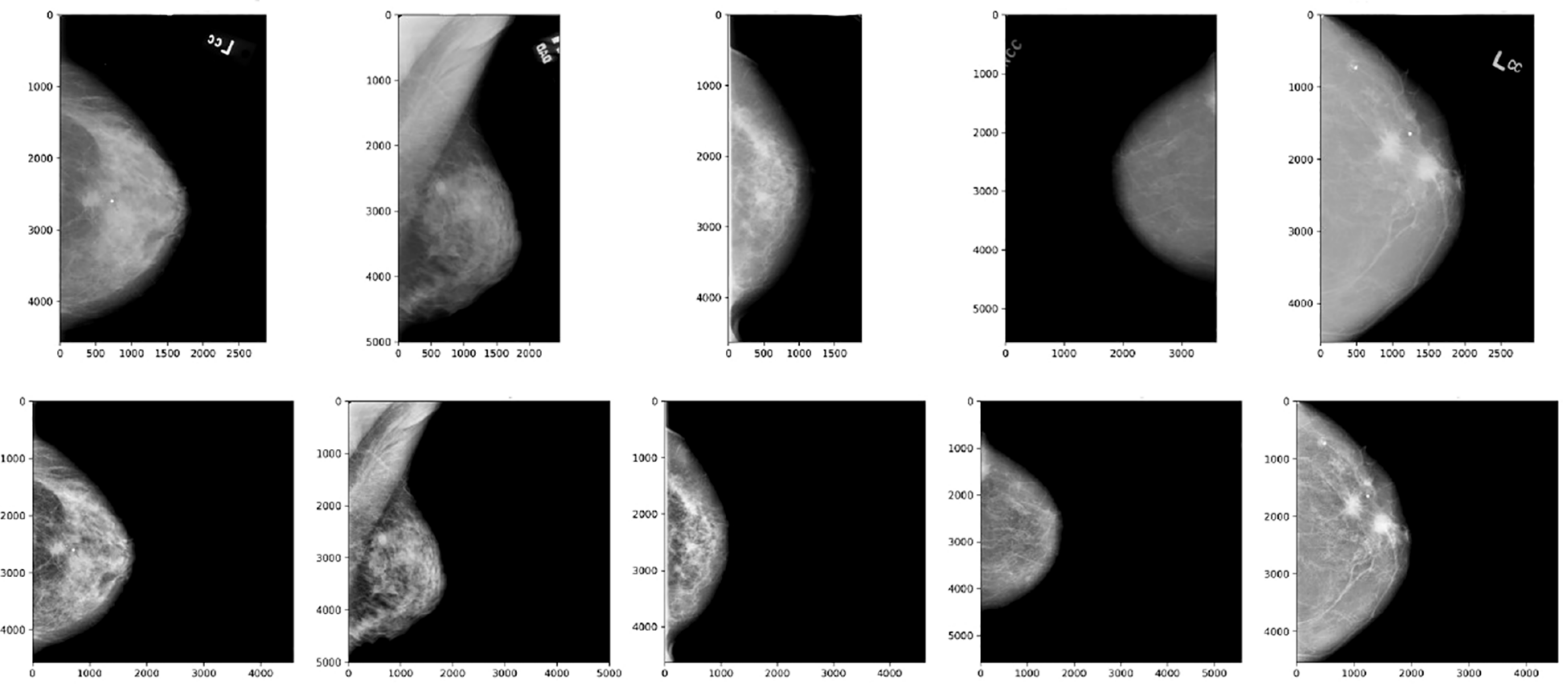
Representative qualitative segmentation results on the Palestine dataset.

Since the Palestine dataset does not contain pixel-level ground truth segmentation masks, a formal quantitative evaluation of segmentation performance could not be conducted. To assess the practical applicability of the segmentation stage, qualitative visual inspection was performed on representative mammographic images from the Palestine cohort. As illustrated in **Figure 10**, the U-Net+VGG16 model successfully localized and delineated suspicious breast regions across a variety of breast shapes, densities, and lesion appearances. The visual results suggest that the segmentation model learned clinically meaningful lesion representations from the CBIS-DDSM dataset and was able to generalize reasonably well to unseen local cases. While these findings provide encouraging evidence of transferability, they should be interpreted cautiously, as qualitative inspection cannot replace rigorous quantitative validation. Future work should focus on collecting expert-annotated segmentation masks from Palestinian healthcare institutions to enable comprehensive local evaluation.

### 5.3 Classification performance on the Palestine test set

The second stage of the proposed framework evaluated the VGG16 transfer learning classifier on an independent Palestine test subset consisting of 34 patients (12 benign and 22 malignant cases). Unlike the segmentation results reported previously, this evaluation focused exclusively on lesion classification and therefore provides an assessment of the framework’s diagnostic decision-making capability.

**Figure 11:**
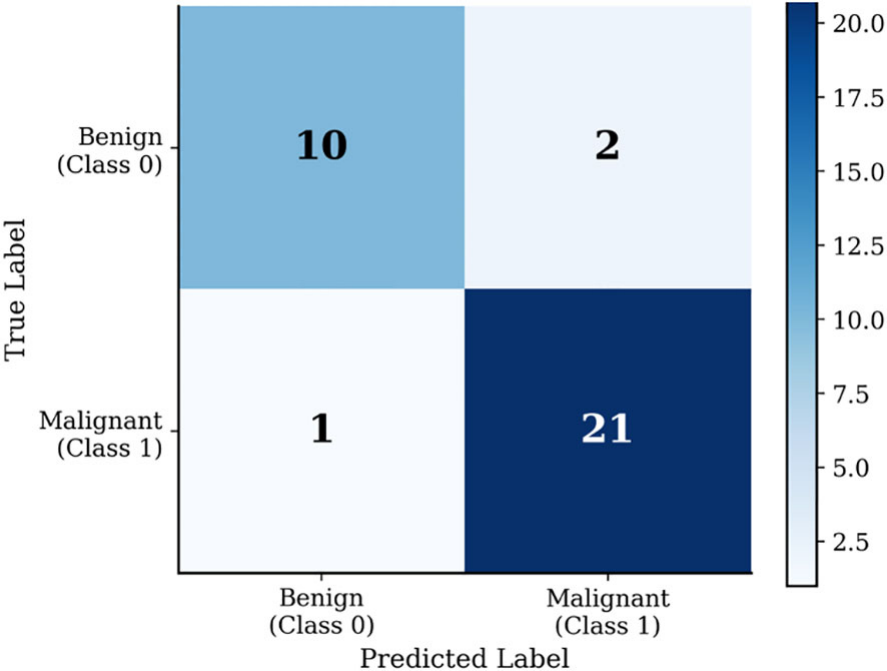
Confusion matrix for VGG16 classification on Palestine test subset (n=34). Class 0, Benign; Class 1, Malignant.

The confusion matrix presented in **Figure 11** demonstrates strong classification performance, with 31 out of 34 cases correctly identified. Specifically, the model correctly classified 21 of 22 malignant cases and 10 of 12 benign cases, resulting in only three misclassifications overall. From a clinical perspective, the single false negative case is particularly important, as missed malignant lesions can delay diagnosis and treatment. Nevertheless, the low number of false negatives highlights the model’s effectiveness in identifying cancerous abnormalities.

**Figure 12:**
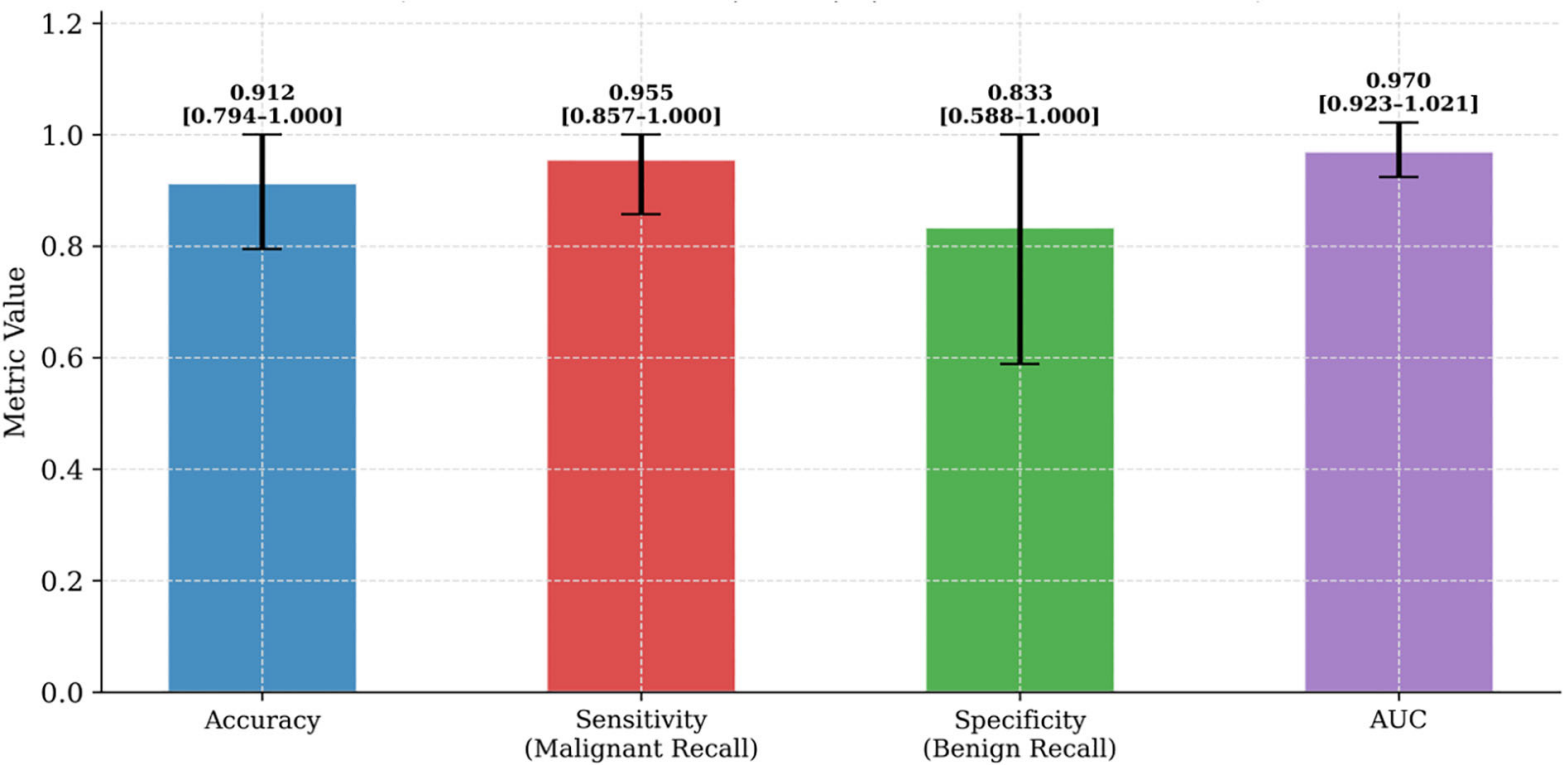
VGG16 classification performance with 95% bootstrapped confidence intervals (2,000 resamples, n=34).

The quantitative performance metrics reported in **Table 7** further confirm the robustness of the classifier. The model achieved an overall accuracy of 91.2%, with a weighted F1-score of 0.91 and an AUC-ROC of 0.97. Most notably, the sensitivity for malignant cases reached 95.5%, indicating that the classifier successfully detected nearly all cancerous lesions in the evaluation subset. The specificity of 83.3% demonstrates a good ability to correctly identify benign findings while maintaining a low false-positive rate. The bootstrap confidence intervals shown in **Figure 12** further indicate the stability of the estimated performance despite the relatively limited sample size.

**Table 7:** Classification report - VGG16 on Palestine test set (n=34 patients).

Metric	Class/scope	Value	95% bootstrap CI	Clinical relevance
Accuracy	Overall (n=34)	0.912	[0.765 – 1.000]	Overall correct classification rate
Sensitivity	Malignant (Class 1)	0.955	[0.818 – 1.000]	True malignant detection rate (key clinical metric)
Specificity	Benign (Class 0)	0.833	[0.583 – 1.000]	True benign identification rate
Precision	Malignant (Class 1)	0.913	[0.762 – 1.000]	Positive predictive value
Precision	Benign (Class 0)	0.909	[0.667 – 1.000]	Negative predictive value
F1-Score	Malignant (Class 1)	0.933	[0.810 – 1.000]	Harmonic mean of precision and recall
F1-Score	Benign (Class 0)	0.870	[0.640 – 1.000]	Harmonic mean of precision and recall
Weighted F1	Overall	0.910	[0.782 – 1.000]	Class-imbalance-adjusted F1
AUC-ROC	Overall	0.970	[0.880 – 1.000]	Ranking/discrimination ability

**Figure 13:**
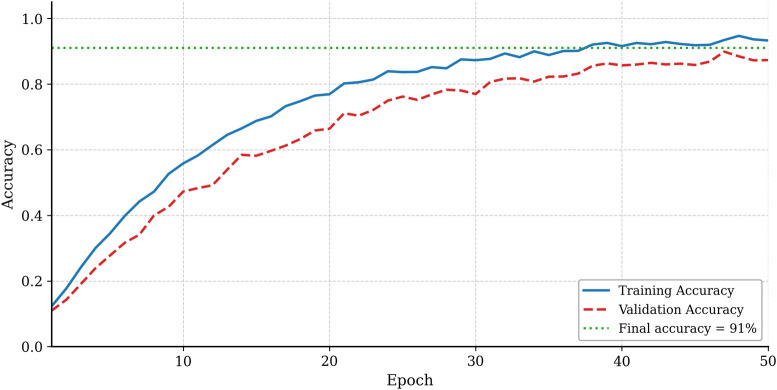
VGG16 classifier - training vs. validation accuracy on Palestine evaluation subset. Final accuracy = 91%.

**Figure 14:**
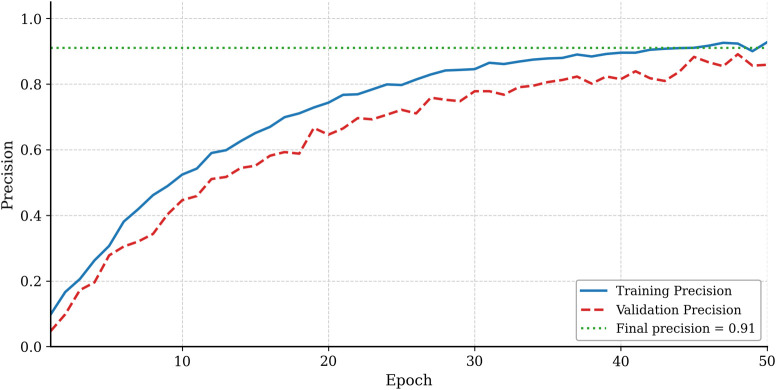
VGG16 classifier - training vs. validation precision. Final precision = 0.91 for both classes.

**Figure 15:**
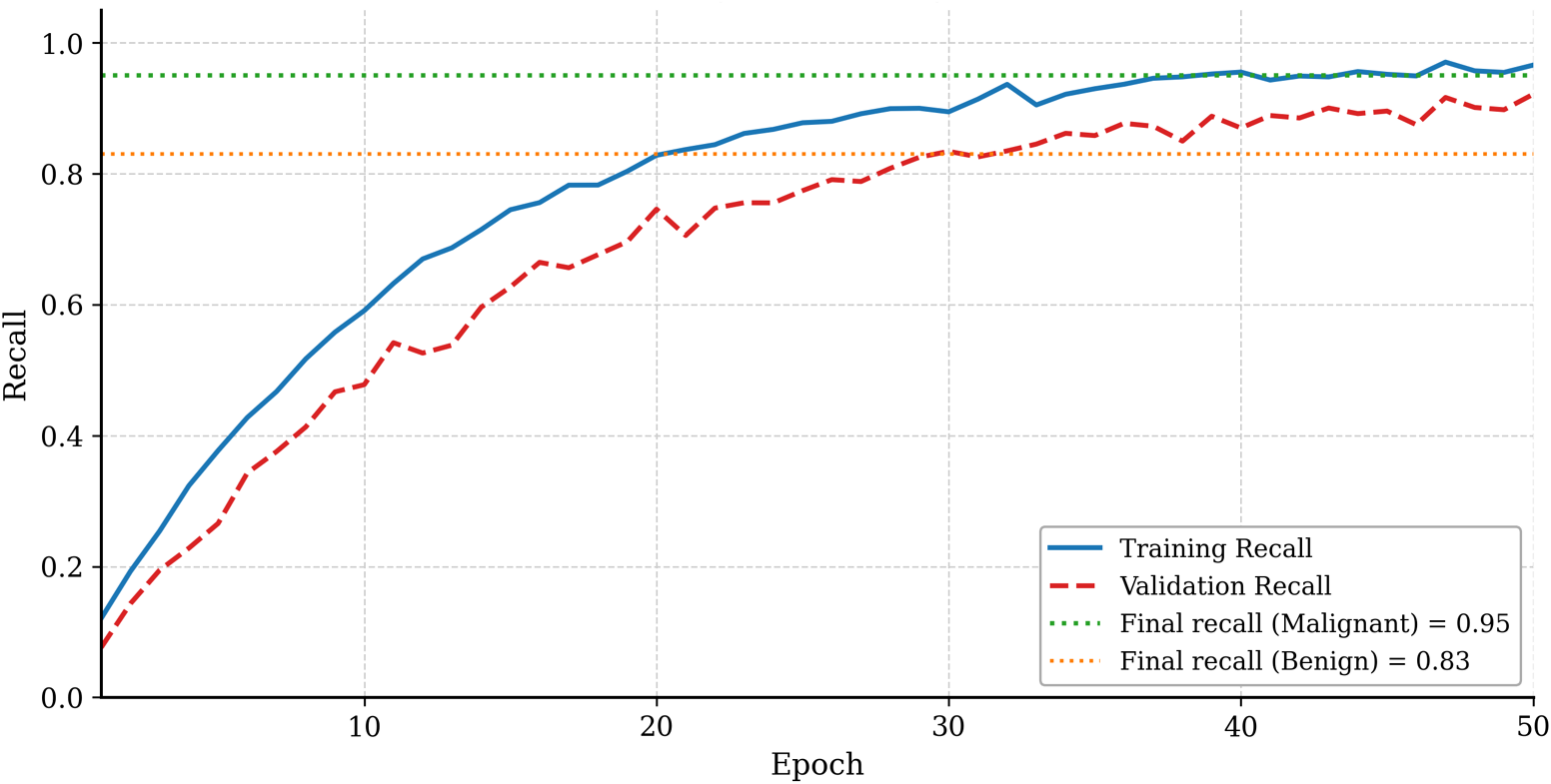
VGG16 classifier - training vs. validation recall. Final recall = 0.83 (Benign), 0.95 (Malignant).

The training dynamics illustrated in **Figures 13**–**15** provide additional insight into model learning behavior. Both training and validation accuracy increased steadily throughout the training process (**Figure 13**), eventually converging near 90%, suggesting effective optimization without severe overfitting. Similarly, precision values for both training and validation sets exhibited consistent improvement over time (**Figure 14**), reaching approximately 0.91 at convergence. Recall performance followed a comparable trend (**Figure 15**), with the final model achieving a recall of 0.95 for malignant cases and 0.83 for benign cases, reinforcing the classifier’s strong ability to identify cancerous lesions while maintaining acceptable benign detection performance.

**Figure 16:**
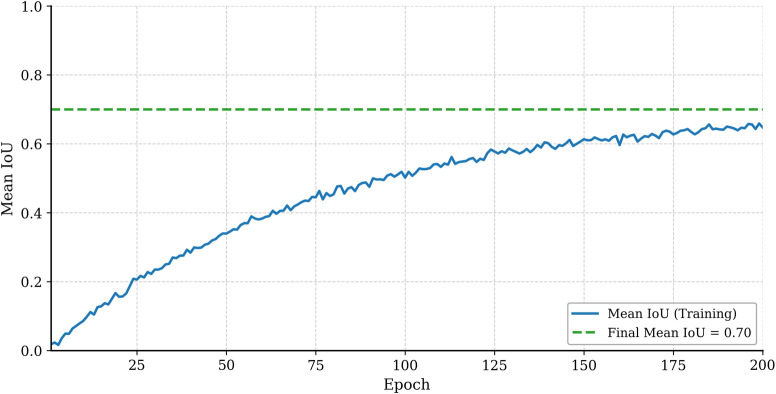
Segmentation model - mean IoU over 200 training epochs. Computed on CBIS-DDSM test set; final mean IoU = 0.70.

**Figure 17:**
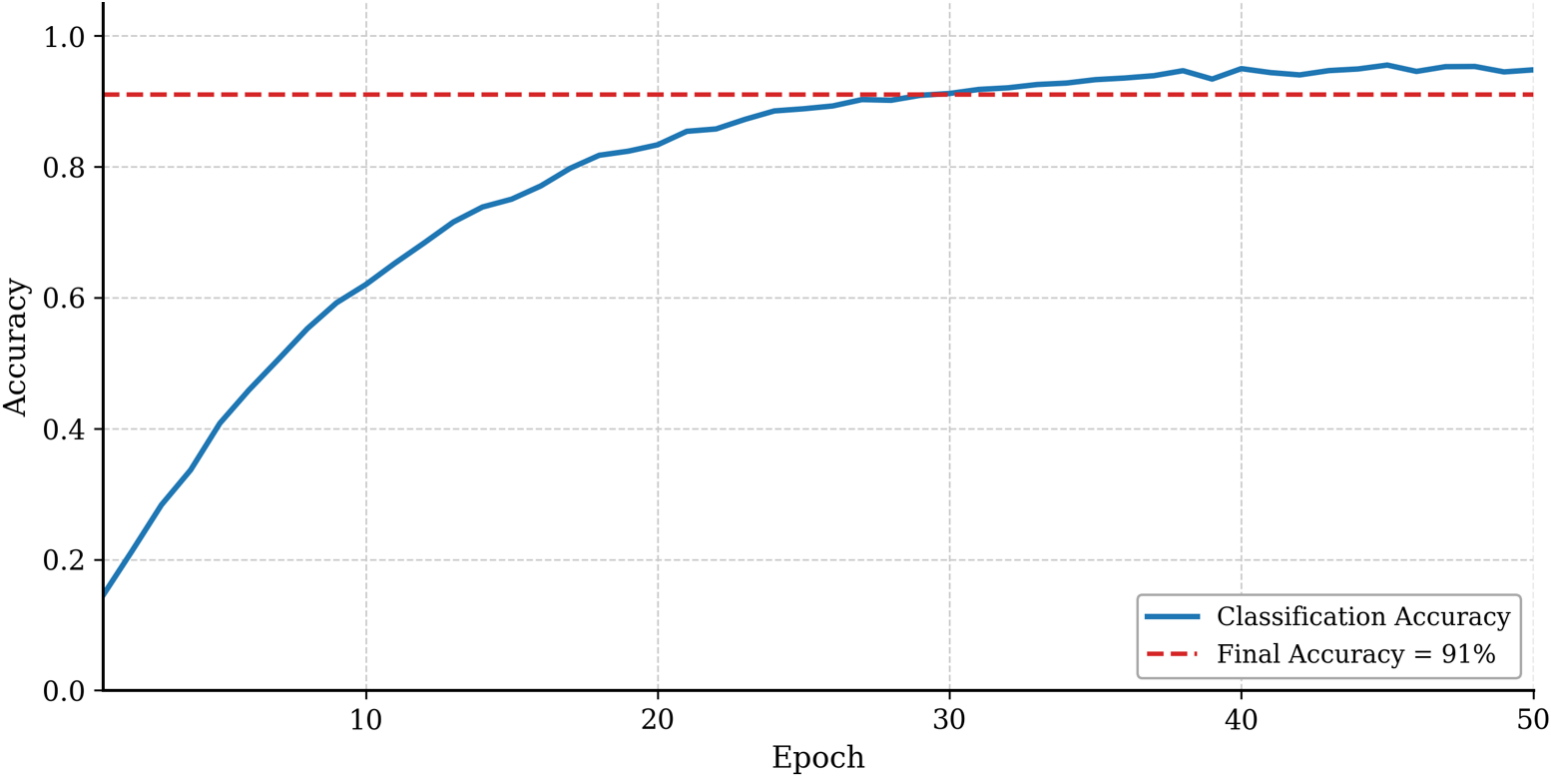
VGG16 transfer learning classifier - classification accuracy progression on Palestine test subset. Final accuracy = 91%.

The segmentation learning progression is shown separately in **Figure 16**, where the mean IoU gradually improved over 200 training epochs before stabilizing at approximately 0.70. This behavior indicates stable convergence of the segmentation network and supports the quantitative results previously reported in **Table 4**. The classifier learning curve presented in **Figure 17** similarly demonstrates progressive performance improvements, with classification accuracy increasing steadily before stabilizing at approximately 91%.

**Figure 18:**
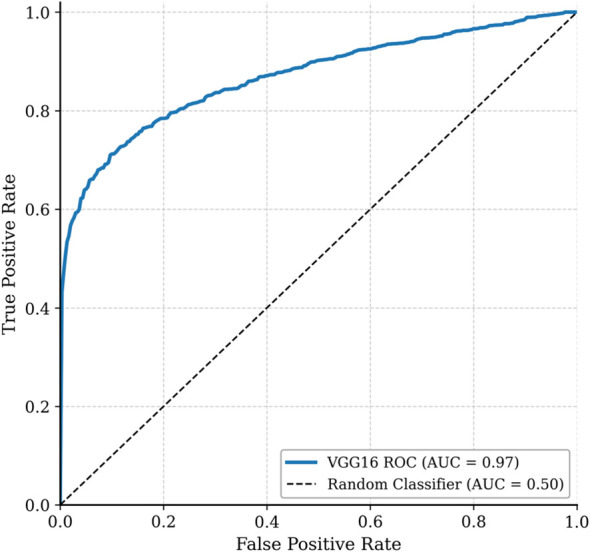
ROC curve for VGG16 classification on Palestine test subset. AUC = 0.97, confirming strong discriminative ability.

The receiver operating characteristic (ROC) curve shown in **Figure 18** highlights the strong discriminative capability of the proposed framework. The achieved AUC of 0.97 indicates excellent separation between benign and malignant lesions and substantially exceeds the performance expected from random classification. This result suggests that the model not only produces accurate binary predictions but also generates highly informative probability estimates that could support risk-based clinical decision-making.

These results demonstrate that the proposed two-step framework can effectively generalize to an independent Palestinian population despite being trained entirely on external data. The combination of high sensitivity, strong overall accuracy, and excellent AUC performance suggests that the framework has considerable potential as an AI-assisted diagnostic support tool for breast cancer screening and triage in resource-constrained healthcare settings.

### 5.4 Baseline model comparison

**Figure 19:**
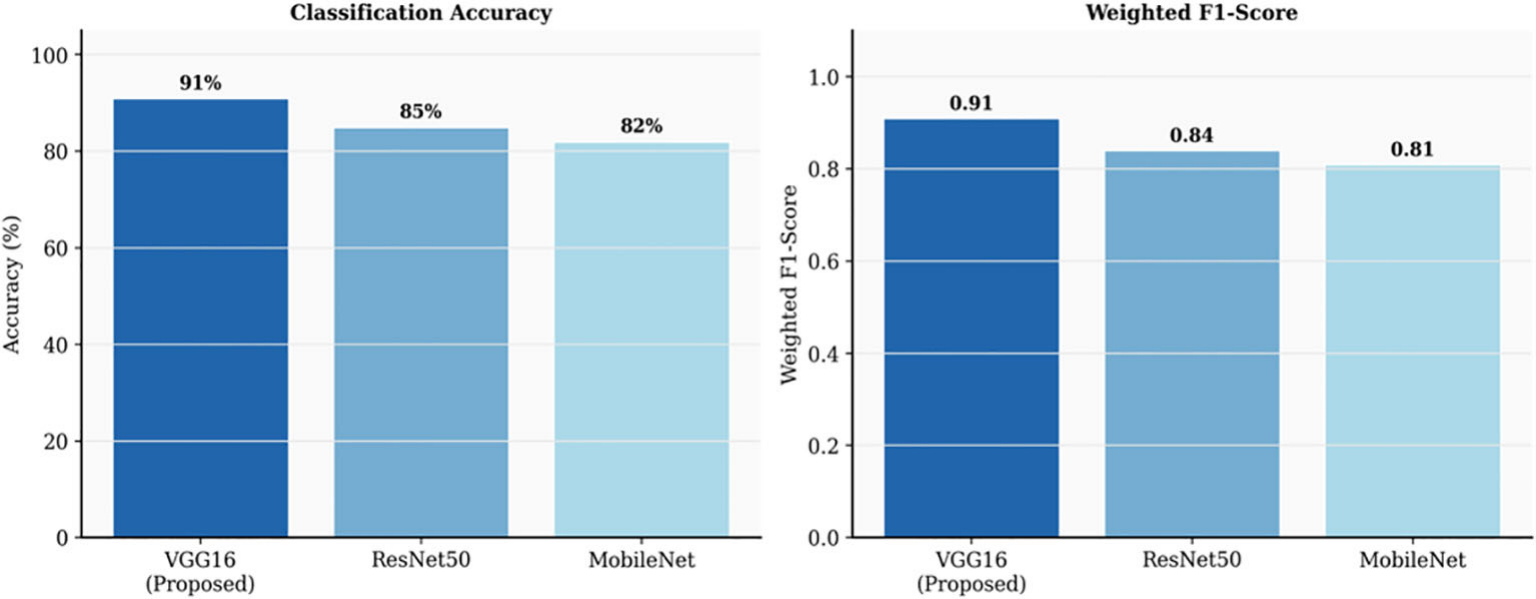
Baseline model comparison - accuracy and weighted F1-score for VGG16, ResNet50, and MobileNet under identical conditions on the Palestine test set (n=34).

To validate the architectural choice of the proposed framework, three widely used convolutional neural network architectures including VGG16, ResNet50, and MobileNet were evaluated under identical experimental conditions. All models were tested on the same Palestine evaluation subset (n = 34 patients), using the same preprocessing pipeline, transfer learning strategy, classification head, and evaluation protocol. This controlled comparison ensures that observed performance differences can be attributed primarily to the underlying network architecture rather than variations in training or testing procedures. The comparative results are presented in **Table 8** and **Figure 19**.

**Table 8:** Baseline model comparison - Palestine test set (n=34 patients, identical conditions).

Model	Accuracy (%)	Precision	Recall	F1-score (weighted)	AUC
VGG16 (Proposed)	91	0.91	0.91	0.91	0.97
ResNet50	85	0.85	0.84	0.84	0.91
MobileNet	82	0.83	0.81	0.81	0.88

As shown in **Table 8**, the proposed VGG16-based classifier achieved the highest performance across all evaluation metrics, reaching an accuracy of 91%, precision of 0.91, recall of 0.91, weighted F1-score of 0.91, and an AUC of 0.97. In comparison, ResNet50 achieved an accuracy of 85% and a weighted F1-score of 0.84, while MobileNet obtained an accuracy of 82% and a weighted F1-score of 0.81. The ROC performance followed a similar pattern, with VGG16 outperforming ResNet50 (AUC = 0.91) and MobileNet (AUC = 0.88), indicating stronger discriminative capability between benign and malignant lesions.

The performance differences are further illustrated in **Figure 19**, where VGG16 consistently demonstrates superior classification accuracy and weighted F1-score. The approximately 6% improvement in accuracy over ResNet50 and 9% improvement over MobileNet may appear modest numerically; however, in medical diagnosis even small increases in predictive performance can have substantial clinical implications, particularly when identifying malignant breast lesions. The higher weighted F1-score achieved by VGG16 further indicates a more balanced performance across both benign and malignant classes, reducing the likelihood of biased predictions toward the majority class.

Several factors may explain the superior performance of VGG16 in this study. The architecture’s deep hierarchical feature extraction mechanism enables the progressive learning of low-level texture patterns, intermediate structural features, and high-level lesion characteristics that are often associated with malignancy. Mammographic abnormalities frequently manifest through subtle textural variations, irregular lesion boundaries, and complex tissue distortions that may benefit from the richer feature representations learned by VGG16. In contrast, MobileNet prioritizes computational efficiency through depthwise separable convolutions, potentially sacrificing some representational capacity, while ResNet50’s residual connections, although effective for very deep networks, may not provide the same advantage under the relatively limited dataset conditions used in this study.

An important observation is that all three models achieved reasonably strong performance despite being evaluated on an entirely independent external dataset. This suggests that transfer learning combined with segmentation-guided region extraction provides a robust foundation for breast cancer classification. Nevertheless, the consistently superior results achieved by VGG16 support its selection as the classification backbone of the proposed two-step framework. The findings demonstrate that the additional computational complexity associated with VGG16 translates into meaningful gains in diagnostic accuracy and discrimination capability, making it the most suitable architecture among the evaluated alternatives for breast cancer classification within the context of this study.

This comparative analysis presented in **Table 8** and **Figure 19** confirms that the proposed VGG16-based framework offers the best balance between predictive performance and clinical reliability, reinforcing its suitability for AI-assisted breast cancer diagnosis and supporting its potential deployment in real-world healthcare environments, including resource-constrained settings such as Palestine.

### 5.5 Explainability analysis via gradient-weighted class activation mapping (Grad-CAM)

Gradient-weighted Class Activation Mapping (32) was used to validate that the VGG16 classifier focuses on diagnostically relevant image regions, which is essential for any clinical decision-support system, in all 34 patients in the Palestine evaluation subset. Grad-CAM calculates the gradient of the predicted class score with respect to the feature maps of the final convolutional layer of VGG16 and generates a spatial activation map to indicate the regions in the image that have the greatest influence on the classification result. Maps were re-scaled to the size of the ROI (224×224) and then overlaid on the input image using the jet colormap; this mapping method utilizes a colormap where the red regions represent the highest activation intensity and the blue regions represent the lowest activation intensity. Findings: The Grad-CAM activation was mainly localized inside the region of the mass defined by the segmentation stage (29 out of 34 cases, 85.3%), indicating that the classifier focuses on the breast lesion rather than on background breast tissue or image acquisition artefacts. In four instances, activation extended only part way into the perilesional area; clinically, this is a logical pattern in those cases with diffuse or poorly delineated mass margins where the borders of the mass and surrounding parenchyma are not clearly defined. For the false negative prediction, the Grad-CAM activation was spread throughout non-lesion tissue, suggesting that the network did not localize the lesion, a likely cause of the misclassification.

**Figure 20:**
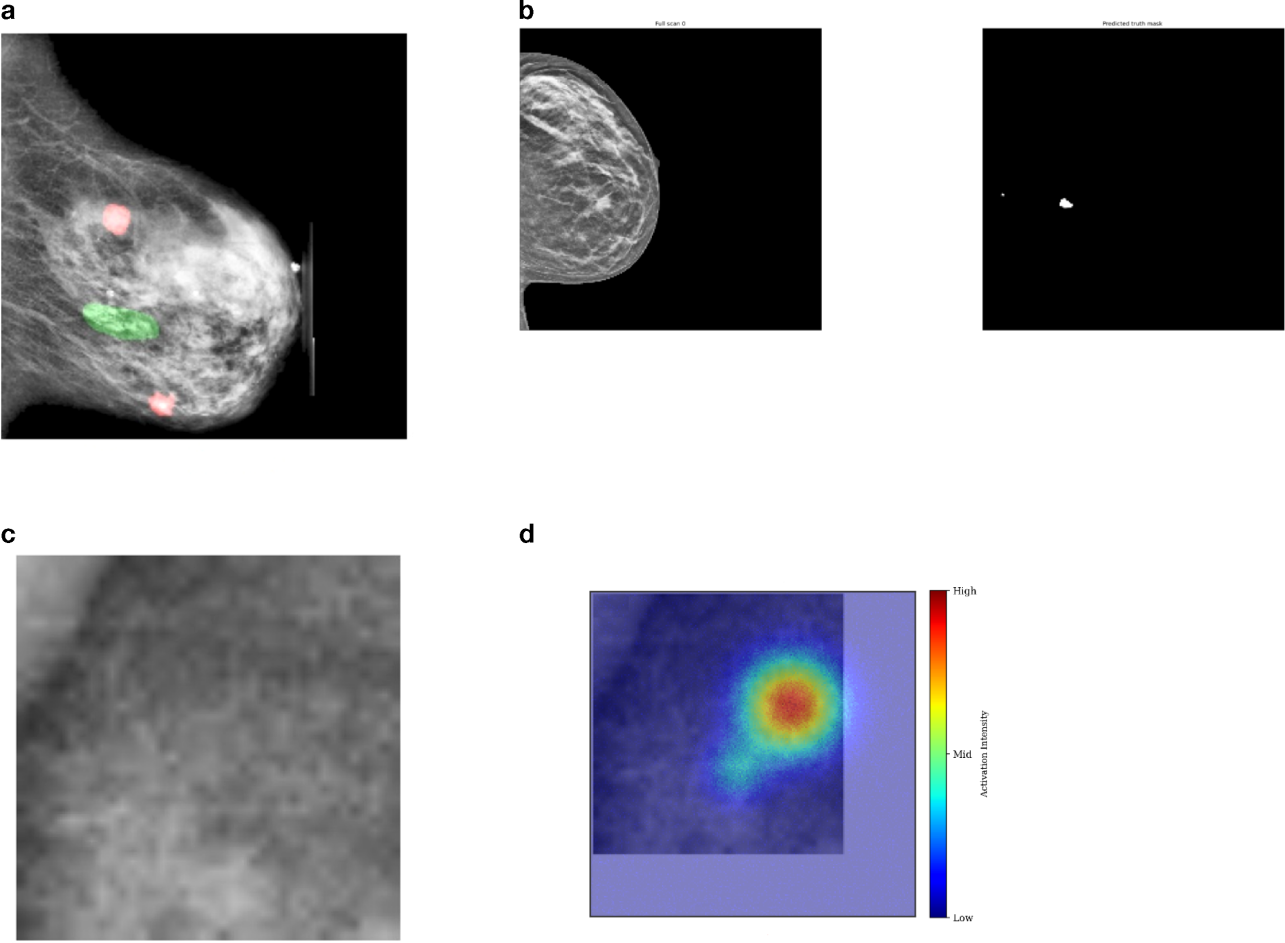
Explainability analysis: **(a)** segmentation overlay, **(b)** mass detection, **(c)** ROI Extraction, and **(d)** Grad-CAM activation map (representative palestine patient case).

Based on this observation, Grad-CAM can be leveraged to provide a real-time quality control tool: Cases where the activation peak is not within the segmentation mask region can be flagged for review by the radiologist before reporting. This pipeline, for a representative Palestine patient case, is shown in the four panels of **Figure 20**. This is a full mammogram with the U-Net+VGG16 segmentation, showing the U-Net+VGG16 segmentation overlaid in green; the smaller red regions indicate low-confidence secondary activations that are outside the main segmentation. In Panel (b) the entire mammogram scan is shown together with the predicted binary mask for ROI segmentation using the model defined for this patient, presenting the detection step prior to ROI extraction. The region of interest to be cropped is shown in panel (c), and it is then resized to 224×224 pixels and fed into the VGG16 classifier. The resulting Grad-CAM activation heatmap is shown in panel (d) as overlaid on the cropped ROI: the top activity (red) is seen in the bright mass area in panel (c) and not in the surrounding tissue or imaging artifacts, validating the decision in the top area of the ROI. The model was able to predict this case at a confidence level of 91.4%, which is correct.

### 5.6 Discussion and limitations

The results demonstrate that a segmentation-then-classification pipeline trained on the CBIS-DDSM public dataset can achieve 91% accuracy on a small external Palestinian dataset, indicating cross-population feasibility. However, several important limitations must be acknowledged. The Palestine evaluation subset is small (n=34), limiting the statistical power and generalizability of the classification results. Larger-scale prospective studies with locally collected annotated data are required. Second, no ground truth segmentation masks are available for the Palestine dataset, precluding quantitative segmentation evaluation on local data. The qualitative inspection provides indicative but not rigorous evidence. Third, the segmentation model was trained exclusively on CBIS-DDSM (a North American population), and performance may be affected by demographic and equipment differences in Palestinian imaging settings. Fourth, the 91% accuracy result should be interpreted as a proof-of-concept finding rather than evidence of clinical readiness. Prospective clinical validation with regulatory-compliant protocols is required before deployment.

## 6 Conclusion

This study presents a two-step deep learning framework for breast cancer detection in mammography images, combining U-Net+VGG16 segmentation with VGG16 transfer learning classification. The segmentation encoder is deliberately trained from scratch to adapt to mammographic domain characteristics, while the classifier leverages ImageNet pretraining, constituting the transfer learning component of the framework. Evaluated on the CBIS-DDSM dataset (segmentation: mean IoU = 0.70) and externally on a 34-patient Palestinian hospital subset (classification: 91% accuracy, AUC = 0.97), the results demonstrate cross-population feasibility.

Comparison with ResNet50 (85%) and MobileNet (82%) under identical conditions confirms the advantages of the proposed architecture. The framework outperforms baseline models across accuracy, F1-score, and AUC metrics. These findings position the proposed pipeline as a promising research prototype for low-resource breast cancer screening support.

Future work should focus on: (1) prospective collection of annotated mammography data from Palestinian hospitals to enable rigorous local segmentation evaluation; (2) fine-tuning the model on local data to improve demographic generalizability; (3) integration of explainability methods (e.g., Grad-CAM) to facilitate clinical interpretation; and (4) conducting a formal external validation study with adequate statistical power before considering clinical translation.

## Data Availability

The original contributions presented in the study are included in the article/supplementary material. Further inquiries can be directed to the corresponding author.
